# Method Validation for the One-Month Stability Study of *trans*–Resveratrol in Human Plasma

**Published:** 2013-05-04

**Authors:** Milad Iranshahy, Amir Hooshang Mohammadpoor, Mohammad Hassanzadeh-Khayyat, Mehrdad Iranshahi

**Affiliations:** 1 Biotechnology Research Center, Mashhad University of Medical Sciences, Mashhad, IR Iran; 2 Department of Pharmacognosy, School of Pharmacy, Mashhad University of Medical Sciences, Mashhad, IR Iran; 3 Department of Pharmacodynamy and Toxicology, School of Pharmacy, Mashhad University of Medical Sciences, Mashhad, IR Iran; 4 Department of Medicinal Chemistry, School of Pharmacy, Mashhad University of Medical Sciences, Mashhad, IR Iran

**Keywords:** Resveratrol, Stability, Antioxidants

## Abstract

**Background:**

*trans*-Resveratrol (t-Res) is a natural phenolic compound with various biological activities such as antioxidant, anticancer, cancer chemopreventive, cardioprotective, and neuroprotective.

**Objectives:**

There is no comprehensive study about the stability of t-Res in human plasma. With respect to extensive use of t-Res in clinical trials and pharmacokinetic studies, in this study, we aimed to measure the plasma stability of t-Res at different conditions of lighting, pH, and temperature, and in the presence of routine antioxidant agents (BHT and VIT C).

**Materials and Methods:**

t-Res stability in human plasma was evaluated at different conditions of lighting, pH and temperature, and in the presence of routine antioxidant agents (BHT and VIT C), and quantitatively determined using HPLC-UV method at 306 nm.

**Results:**

Our findings revealed that t-Res was quite stable in different temperatures ranging from -70 °C to 25 °C, and acidic pH conditions in plasma for a month, when protected from light, but it was unstable in alkali pH and in lighting conditions.

**Conclusions:**

In lighting conditions, most of t-Res was isomerized to the other isomer cis-resveratrol. t-Res is unstable in alkali pH and when exposed to light. t-Res was quite stable at other conditions, and several freeze-thaw cycles did not have any effect on its stability.

## 1. Background

**Figure 1.**
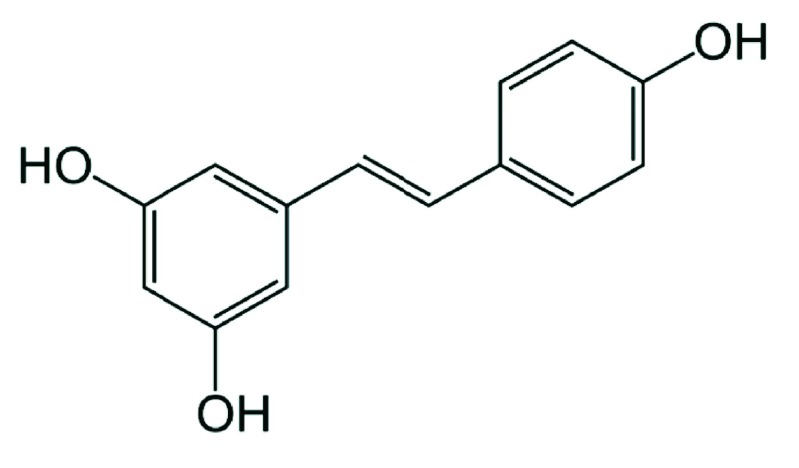
The Chemical Structure of *trans* – Resveratrol

t-Res (3, 4, 5-trihydroxy-trans-stilbene) is a natural phenolic compound with various biological activities such as antioxidant (1), cancer preventive (2-4), cardioprotective (5), phytoestrogenic and neuroprotective (6, 7) (Figure 1). Most of these biological effects are reported from t-Res (8, 9). *Cis* - Resveratrol is an artificial compound which is produced from t-Res, when t-Res is exposed to light. t-Res as a phytoalexin appears in many foods such as grape seeds, wine, soybean and peanut. No *cis*-resveratrol has been detected from natural sources. *Polygonum cuspidatum* is one of the richest sources of t-Res (10). t-Res is known responsible for French paradox (11). t-Res, one of the major components of red wine, showed high cardioprotective effects (12). Several mechanisms were reported for cardioprotective activity of t-Res such as inhibition of platelet aggregation (13), and antioxidant effects on cholesterol metabolism (5). Most of studies had been performed in vitro, and more studies on human volunteers are necessary (14).

In organic solvents, t-Res was unstable in alkali pH and in front of light. Brent C. et al. reported that t-Res is susceptible to degradation in alkali pH. In laboratory lighting conditions, an equilibrium (after conversion of *trans* - Resveratrol to *cis* - resveratrol and in favor of *cis* form) occurred between *cis* and *trans* - Resveratrol (15).

## 2. Objectives

In spite of a few reports on the stability of t-Res in organic solvents, no study has been conducted on t-Res stability in human plasma to date. Since the difference of plasma environment and organic solvents, there is a need to know whether the stability of t-Res is affected by proteins, enzymes or other factors in plasma (decompose / protect). Besides, a large number of researchers have used plasma for extraction and quantification of t-Res in their pharmacokinetic studies. The questions are how long we can keep plasma with a few or no changes in the content of t-Res, and whether freezing-thawing or acidification (that is normally used for extraction of t-Res from plasma) of plasma affects the stability of t-Res. In addition, no study has been conducted on the stability of this compound in a long period of time. Because of the limited information on this area, we decided to study the stability of t-Res in plasma at different conditions for a month.

## 3. Materials and Methods

### 3.1. Chemicals

t-Res, butylated hydroxyl toluene (BHT) and vitamin C (VIT C) were purchased from Sigma-Aldrich Chemical Co. Human plasma was obtained from healthy volunteers from Iranian Blood Transfusion Organization. All solvents were purchased from Mojallali Co. (Iran), redistilled and filtered before use. Acetate cellulose and polyamide filters (0.45 µM) were obtained from Albet LabScience and Whatman companies, respectively.

### 3.2. Instrumentation and Chromatographic Conditions

Chromatography, high pressure liquid (HPLC) analysis was performed on an analytical Knauer HPLC system equipped with k-1001 Knauer pump (flow rate 1 mL/min), C18 hypersil column and k-2600 Knauer UV detector (λ_max_ = 306 nm). Chromatographic separation was accomplished by injecting the sample onto a C18 hypersil column 4.6 × 250 mm. An isocratic elution was carried out with the solvent system methanol: water: acetic acid (60:38:2). Injection volume was 40 μL.

### 3.3. Sample Preparation

Stock solution of 2 mg/mL t-Res was prepared in ethanol (40% v/v). Subsequently, the concentrations 0.02, 0.05, 0.1, 0.5, and 2 μg/mL of t-Res were prepared from the stock. The ethanol solutions of t-Res were only used for the preparation of a standard curve. To determine the stability of t-Res in plasma, the concentrations 0.5, 1, and 2 μg/mL of t-Res were prepared in plasma according to the following conditions. To prepare acidic and neutral plasma samples, phosphoric acid 85% (0.098 g in 90 cc plasma) and NaH_2_PO_4_ (0.12 g in 90 cc plasma) were used, respectively. HCl was used to lower the pH, and NaOH was used to raise the solution pH. To prepare higher pH values (pH = 12), ethanolamine (0.061 g) was added in plasma (90 cc plasma). To determine the effect of antioxidant agents on the stability of t-Res in plasma, the concentration 0.5 mg/mL of VIT C and BHT was applied in t-Res solutions. t-Res solutions were also subjected to different temperature conditions (25, -20 and -70 ^°^C) and photodegradation in front of light (Except this group, the other groups of t-Res solutions were protected from light). Finally, we had nine groups of each concentration of t-Res (0.5, 1 and 2 μg/mL, totally 27 samples) which were tested in triplicate. The stability of t-Res in each sample was tested during one month storage. To extract t-Res from plasma, 500 µL of the solvent system methanol: water: acetic acid (80:17.5:2.5) was added to 500 µL plasma and agitated in the vortex for 30 seconds. Afterwards, all samples were centrifuged at 10000 rpm for 10 min at room temperature, and supernatant were analyzed by HPLC as described.

### 3.4. Method Validation

The extraction methods were validated according to previous studies with a small change in solvent system (16). The solvent of extraction was methanol: water: acetic acid (80:17.5:2.5). The criteria of our method including precision and accuracy, recovery, sensitivity, linearity, and selectivity were evaluated as in the following.

### 3.5. Precision and Accuracy

The inter-day and intra - day precisions of the analytical method were determined by assaying five samples at five concentrations ranging from 0.02 to 2 μg/mL. The inter - day precision was determined by analyzing samples on three different days. The intra - day precision was determined by analyzing samples within a day. Accuracy of method was measured by Formulae 1 and 2 in which calculated concentration was obtained from calibration curve.

**Formula 1.** *Bias* = Calculated – True conc. / True conc.

**Formula 2.** *Accuracy* = Bias% + 100

### 3.6. Recovery

The recovery of t-Res from plasma was determined by spiking blank samples at the final concentrations of 0.02, 0.05, 0.5, 1 and 2 μg/mL.

*Recovery* = AUC Plasma / AUC Ethanol

Where AUC plasma is the mean value of the area under the curve (AUC) of t-Res HPLC chromatogram peak obtained from the plasma sample, and AUC ethanol is the mean value of the AUC of t-Res HPLC chromatogram peak obtained from the ethanol 40% sample.

### 3.7. Sensitivity

The limit of detection (LD) and the limit of quantification (LQ) were determined by measuring the analytical background response, and running plasma samples. The signal-to-noise ratio was used to determine the LD and LQ. LD and LQ were considered to be 3 and 10 times, superior to the baseline noise analyzed using the maximum sensitivity allowed by the system respectively.

### 3.8. Linearity

Spiked plasma samples that contained increasing concentrations of t-Res: 0.02, 0.05, 0.1, 0.5, and 2 μg/mL were analyzed according to the above procedure. Integrated peak areas were plotted against t-Res concentration, and linear regression was performed by the least squares methods.

### 3.9. Statistical Analysis

Data were analyzed by one-way ANOVA and Tukey’s test (SPSS version 11.5). A P value less than 0.05 was considered as significant. Data were expressed as the Mean ± SD. All concentrations were expressed in μg/mL plasma.

## 4. Results 

### 4.1. Method Validation

#### 4.1.1. Linearity

Five calibration runs of spiked plasma were performed and were linear over the measured range. The calibration curve was defined as the following equation y = 1.772x + 0.0128; r ^2^ = 0.9995.

#### 4.1.2. Selectivity

**Figure 2.**
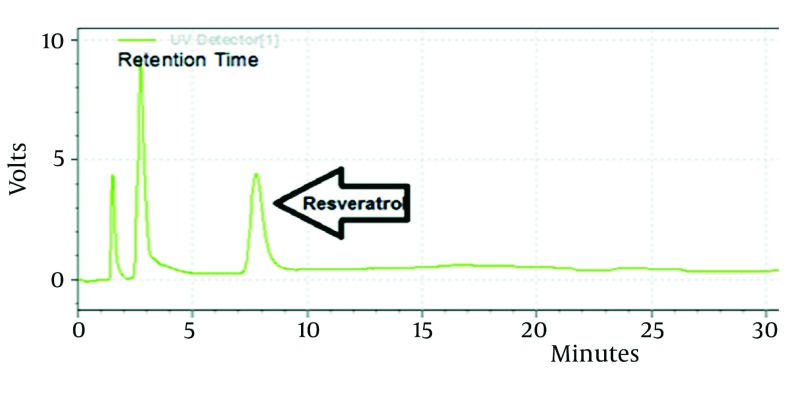
HPLC Chromatogramof a Plasma Containing t-Res (0.1 µg/mL) at 306 nm

No interferences were from endogenous compounds at the retention time of t-Res comparing with blank plasma (Figure 2).

#### 4.1.3. Sensitivity, Accuracy and Precision

The LD and LQ for t-Res (signal - to - noise ratio of 3 and 10, respectively) were 0.006 and 0.02 μg/mL in plasma solution, respectively. Accuracy of the method was shown in Table 1. The intra-day precision of the assay was evaluated by performing 5 replicate injections of 2, 0.5, 0.1, 0.05, and 0.02 μg/mL of t-Res in plasma. The coefficients of variation were 9.21%, 12.87%, 10.78%, 13.14%, and 7.62%, respectively. The inter - day precision of the assay was also evaluated by performing 5 replicate injections of 2, 0.5, 0.1, 0.05, and 0.02 μg/mL of t-Res in plasma and the coefficients of variation were 4.2%, 9.12%, 2.5%, 12.95%, and 8.23%, respectively.

**Table 1.** Accuracy and Bias Percentage of This Method for Five Various Concentrations of *trans* - Resveratrol in

Accuracy	Bias, %	Calculated Conc., µg/mL	True Conc., µg/mL
**99.5**	-0.5	1.99	2
**104**	4	0.52	0.5
**90**	-10	0.09	0.1
**88**	-12	0.044	0.05
**105**	5	0.021	0.02

#### 4.1.4. Recovery

Recoveries were measured for five replicate injections. Extraction recoveries from plasma in the concentrations of 0.02, 0.05, 0.1, 0.5, and 2 μg/mL were 74.6%, 75.4%, 80.8%, 86.9%, and 84.9%, respectively.

## 5. Discussion

**Figure 3.**
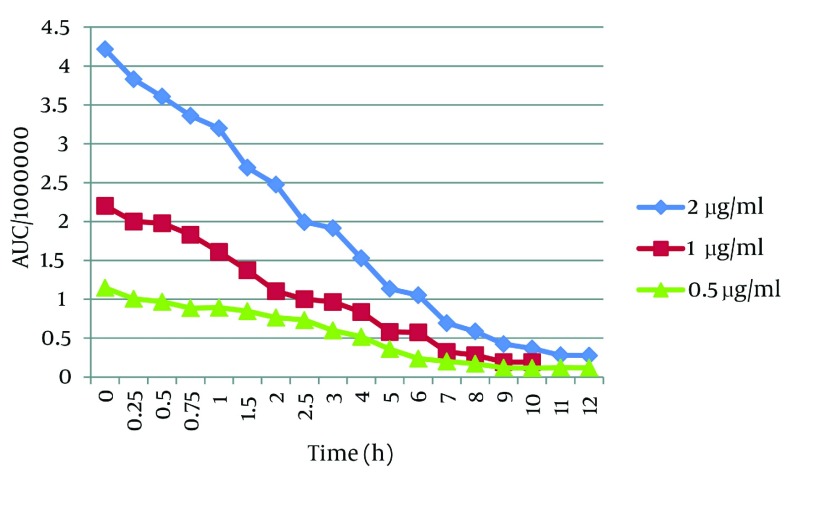
The Plasma Stability of *trans* - Resveratrol Over the Time Exposed to Light

When the effect of lighting conditions on the stability of t-Res was studied, we observed that t-Res was unstable (Figure 3). This result was in agreement with the literature (12, 16) reporting that t-Res was converted to *cis* - resveratrol when exposed to light. In fact, there is a balance between *cis* and *trans* forms in the favor of *cis* form, when exposed to UV (366 nm) or light (7). As shown in Figure 3, t-Res was susceptible to degradation and its half - life was 2 h, approximately. After 10 hours, all t-Res was decomposed, and only a small quantity was detectable in plasma (Figure 3). This half - life is more than the half - life of t-Res in organic solvents at lighting conditions. To justify this difference, we have to consider the other elements of plasma that may interfere in this case. t-Res showed a strong affinity towards protein binding at physiological conditions (17). It means that most of t-Res is bound with proteins. It has been proved that chemical instability of t-Res changed when encapsulated into liposomes containing albumin.

The interaction of t-Res with proteins and other large molecules prevents t-Res from *cis* / *trans* racemization (18). The mentioned study showed that lipids also protected t-Res under UV light exposure, with 70% t-Res still present after 16 min of UV light exposure compared to 10% when t-Res was exposed in the free form (18).

**Figure 4.**
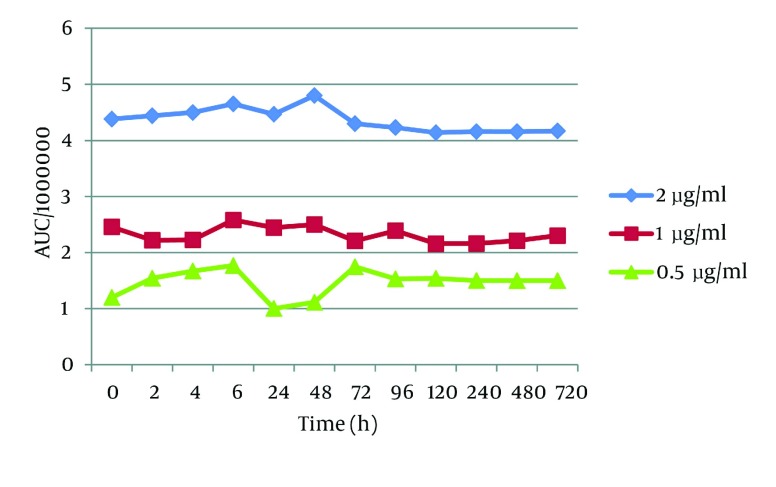
The Plasma Stability of *trans* - Resveratrol Over the Time in -70 °C

To determine the stability of t-Res in the other conditions, we protected plasma samples from light. t-Res was quite stable in different temperature conditions. Considering frozen samples at -20 and -70^°^C, our findings revealed that several freeze-thaw actions did not have any effect on the stability of t-Res, and the samples were quite stable for a month (Figure 4).

**Figure 5.**
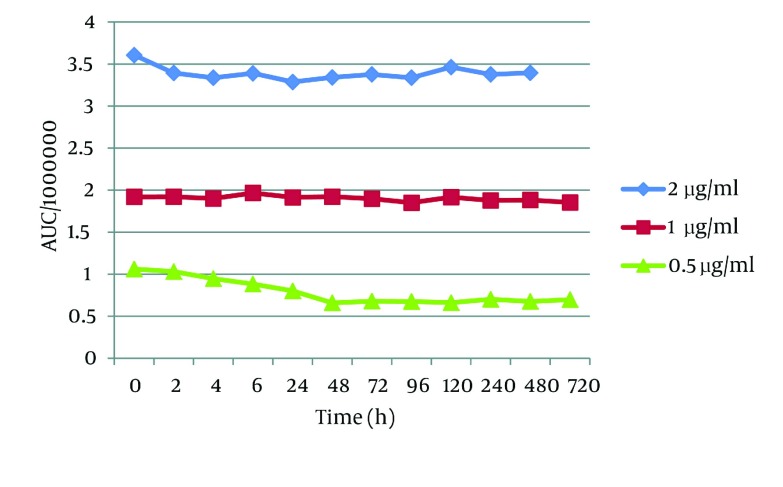
The Plasma Stability of *trans* - Resveratrol Over the Time in pH = 3

**Figure 6.**
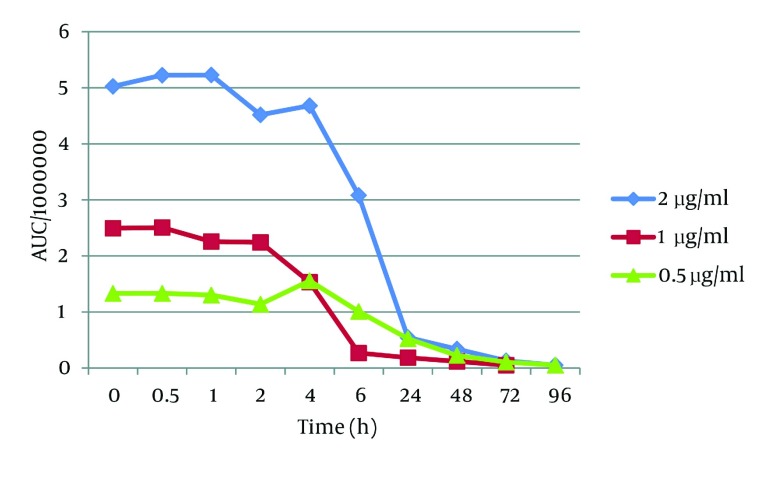
The Plasma Stability of *trans* - Resveratrol Over the Time in pH = 12

**Figure 7.**
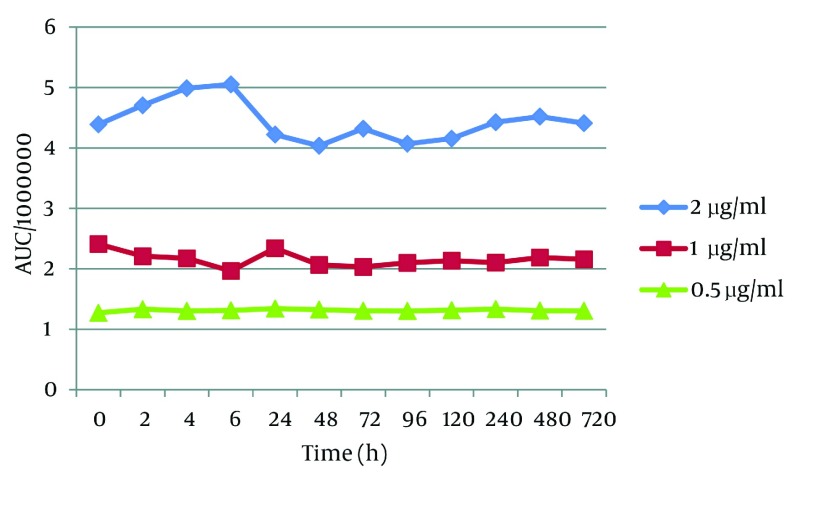
The Plasma Stability of *trans* - Resveratrol Over the Time in the Presence of BHT

When protected from light, t-Res was stable for 720 hours (one month) in pH = 3 (Figure 5) and 7 (data was not shown). In contrast, t-Res was unstable in pH 12 with half - lives of 12, 10 and 20 h for the concentrations 2, 1 and 0.5 μg/mL, respectively (concentration - dependent, Figure 6). However, these half - lives are much more than that previously reported in the literature (the stability of t-Res in buffer pH = 10 with the initial half - life 1.6 h) (7). This difference between the plasma stability of t-Res and its stability in non-physiological conditions such as organic solvents may be also related to the high affinity of t-Res to plasma proteins, as mentioned previously.

With respect to the stability of t-Res in normal temperature when protected from light, Vit C and BHT did not show any significance difference on the stability of t-Res (Figure 7).

t-Res was quite stable in different temperatures ranging from -70 °C to 25 °C and acidic pH conditions in plasma for a month, when protected from light, but it was unstable in alkali pH and in lighting conditions. Although, the pattern of the chemical instability of t-Res in plasma was similar to that of its chemical instability in organic solvents, however, it seems that t-Res is less unstable in plasma at alkali pH and lighting conditions as compared to organic solvents such as aqueous ethanol.
